# The impact of emotion regulation strategies on disordered eating behavior in children and adolescents with type 1 diabetes: a cross-sectional study

**DOI:** 10.3389/fped.2024.1400997

**Published:** 2024-08-07

**Authors:** Xin Yang, Hui Jiang, Meng Lin, Shuang Yu, Jin Wu

**Affiliations:** ^1^ Department of Pediatric Genetics, Metabolism and Endocrinology Nursing, West China Second University Hospital, Sichuan University, Chengdu, Sichuan, China; ^2^ Key Laboratory of Birth Defects and Related Diseases of Women and Children, Sichuan University, Ministry of Education, Chengdu, Sichuan, China

**Keywords:** emotion regulation, eating disorders, type 1 diabetes, children and adolescents, cross-sectional study

## Abstract

**Objective:**

Difficulties in emotion regulation (DERs) can contribute to disordered eating behavior in children and adolescents with type 1 diabetes (T1D), although it is unknown how DERs may affect eating behavior in these children and adolescents. This study examined the relationship between disordered eating behaviors and emotion regulation in children and adolescents with T1D.

**Methods:**

For this cross-sectional study, 128 patients (aged 8–16 years) were recruited to complete the Diabetes Eating Problem Survey-Revised (DEPS-R) and Difficulties in Emotion Regulation Scale (DERs).

**Results:**

The mean age of the 128 patients (99 females) who completed the DEPS-R was 11.63 ± 2.27 years. The participants' mean DEPS-R score was 17.78 ± 8.56 points. Of the total sample, 61 participants' scores surpassed the established threshold, resulting in a DEPS-R positivity rate of 47.66%. The participants' mean total DERS score was 72.3 ± 21.15 points, and it was found that children and adolescents with T1D who had a positive DEPS-R screening result had significant differences in emotional regulation and that eating behavior disorders were positively correlated with emotional regulation and all dimensions scores.

**Conclusions:**

The prevalence of disordered eating behavior is high among children and adolescents with T1D. DERs are related to disordered eating behavior in children and adolescents with T1D. The novel finding that DERs may be a predictor of eating problems lends preliminary support for the inclusion of DERs in future risk models and as a potential target for intervention.

## Key points

• The prevalence of disordered eating behavior is high among children and adolescents with T1D.
• The influence of emotion regulation on disordered eating behaviors in children and adolescents with T1D should not be underestimated.
• It is crucial for healthcare providers in clinical settings to be attentive to the adverse consequences associated with disordered eating behavior and emotional regulation.
• The regular assessment of disordered eating behavior and emotion regulation in adolescents diagnosed with T1D is pivotal, as is timely intervention to avoid progression toward clinically relevant psychiatric disorders or clinical complications in late adolescence and adulthood.

## Introduction

Type 1 diabetes (T1D) is one of the most common chronic illnesses in children and adolescents, and its prevalence is increasing globally (1). An estimated 98,200 children younger than 15 years are diagnosed with T1D each year, and 600,900 children younger than 15 years worldwide are projected to have this disease (2). In Asia, China ranks first in terms of the number of adolescents with T1D (3, 4). However, T1D management is a complex psychological process that necessitates adhering to a demanding structured plan, comprising a prescribed pharmacological regimen, regular blood glucose monitoring, correct nutritional administration, and proper physical activity (5). In 2022, the International Society for Pediatric and Adolescent Diabetes (ISPAD) clinical practice guidelines on glycemic targets and glucose monitoring for children, adolescents and young adults with diabetes state that the primary goal of diabetes treatment is to achieve adequate metabolic control—that is, to maintain one's blood glucose levels as close to the physiological range as possible—to reduce potential complications (6). The data show that the average glycosylated hemoglobin (HbA1c) level of adolescent T1D patients in China is as high as 9.3%, and the percentage of patients with who meet their glycemic targets (HbA1c level <7.5%) is only 15.5%, which is far lower than of 44%∼59% reported in countries such as the United States and Germany (7). Thus, maintaining a regular, balanced healthy diet is a prerequisite for diabetes management (4).

The requirement for rigorous dietary control, which includes restrictions on sugar and carbohydrate intake, can cause fear and anxiety for many adolescents with T1D, who are in a crucial stage of physical and mental growth (8). Furthermore, adolescence is a period that involves in tense emotional ups and downs, and the diagnosis and treatment of T1D can add to the emotional burden of adolescents (9). Emotional regulation is fundamental for human life, and well-being and emotions are usually balanced. However, this delicate and refined mechanism may sometimes become dysfunctional when negative emotions are not correctly counterbalanced. This imbalance may cause maladaptive behaviors, especially during adolescence (10). In addition, the presence of alexithymia increases the vulnerability of adolescents (11). Adolescents may encounter challenges in controlling their emotions and feel angry, frustrated, anxious, or helpless, leading to irrational responses to managing T1D, such as skipping insulin injections, neglecting blood sugar monitoring, and even binge eating (12). Teenagers may also encounter psychological and social pressures, feel different or isolated from their peers, and lack effective emotional regulation, all of which can further lead to disordered eating behavior (DEB) (13).

DEB is characterized by a cluster of various symptoms and maladaptive behaviors in individuals with diabetes, including worry about eating, restrictive eating, and binge eating, and compensatory behaviors, such as fasting, vomiting, laxative abuse, excessive exercising, and insulin restriction (14). These behaviors are also referred to as subclinical eating disorder symptoms and are not yet at the frequency or severity level to warrant a formal eating disorder diagnosis (15). When T1D and disordered eating coexist, both condition have significant health implications. These effects include inadequate glycemic control comorbidities such as retinopathy, nephropathy, and foot problems; diabetic ketoacidosis; and even death (16).

Overall, DEB is a significant health concern for children and adolescents with T1D. In recent decades, an increasing number of studies have focused on explaining the problem of DEB in children and teenagers with T1D, as well as the repercussions of this combination of these disorders. There are limited scientific investigations regarding the link between the management of emotions and the presence of disordered eating patterns in young individuals with T1D. Moreover, there is a lack of data pertaining to the deficits and facets of emotion regulation in individuals with T1D who DEB. In particularly, a gap in related knowledge is evident within the context of China.

The purpose of this study was to investigate whether children diagnosed with T1D who are at risk for DEB experience challenges in regulating their emotions and, if so, which specific aspects of emotion regulation are affected. Our hypothesis postulated that children with T1D who are at risk for DEB [positive Diabetes Eating Problems Survey-Revised (DEPS-R) screening] encounter substantially greater difficulties in managing their emotions than do those who are not at risk for DEB (negativer DEPS-R screening).

## Methods

### Study design and participants

Children and adolescents with T1D who visited West China Second Hospital of Sichuan University, China, between March 2022 and November 2023 were recruited and consented to participate in this cross-sectional study. To select the participants, a nonprobabilistic convenience sampling method was employed. The inclusion criteria for the patients were as follows: aged 8∼16 years had T1D diagnosed according to the American Diabetes Association criteria were receiving insulin treatment were diagnosed with diabetes at least six months before the study and were fluent in Chinese and able to complete the surveys. The exclusion criteria were as follows: major chronical medical, psychosocial or family issues that would interfere with study participation.

## Instruments

### General demographic characteristics

Data on general demographic characteristics, including height, weight, sex, age, education level of the primary caregiver, per capita household income, and other medical conditions, were collected from the most recent routine diabetes-related visit records. Additional diabetes management information was obtained from electronic medical records. The height and weight of the participants were measured uniformly by standardized trained nurses in the hospital. Body mass index (BMI) percentiles were calculated using the Centers for Disease Control and Prevention growth charts. Adolescents were categorized by weight status according to BMI percentile: underweight (<5th percentile), normal weight (5th to <85th percentile), overweight (85th to <95th percentile) and obese (≥95th percentile) (17).

### Diabetes eating problem survey-revised

The DEPS-R is a tool that was specifically created to assess eating problems in individuals with T1D. This survey is used to evaluate diabetes-specific compensatory behaviors, such as restricting or omitting insulin intake to achieve weight loss (18). This self-administered instrument consists of 16 items that are specifically related to diabetes and are rated on a 6-point Likert scale ranging from 0 (never) to 5 (always), indicating the frequency of the behaviors. Previous studies have suggested a cutoff score of 20 points for the DEPS-R, which was used in this study for comparison. Scores within the range of 10 to 19 points are considered to indicate moderately disordered eating behaviors, warranting further assessment (19). Generally, higher scores indicate more severe pathology. The reliability and validity of the Chinese version of the DEPS-R were confirmed by Lv W (20). The internal consistency of the DEPS-R is deemed satisfactory, as evidenced by a Cronbach's *α* coefficient of 0.85. Additionally, the Pearson correlation coefficient for the DEPS-R was found to be 0.916, and the Kaiser–Meyer–Oklin value was calculated to be 0.702. The survey can be completed within a span of 10 min.

### Difficulties in emotion regulation scale

To assess the adolescents' emotional regulation we utilized the Difficulties in Emotion Regulation Scale (DERS). This extensively utilized 36-item self-report measure, which was created by Gratz and Roemer (21), consists of 6 distinct subscales: (a) the awareness subscale, which addresses the lack of awareness of emotional responses; (b) the clarity subscale, which focuses on the lack of clarity of emotional responses; (c) the non-acceptance subscale, which targets the nonacceptance of emotional responses; (d) the strategies subscale, which examines limited access to effective strategies; (e) the impulse subscale, which explores difficulties in controlling impulsive behavior when experiencing negative affect; and (f) the goals subscale, which investigates difficulties in engaging in goal-directed behavior when experiencing negative affect. In terms of its reliability, the DERS has strong internal consistency (Cronbach's ɑ = 0.93). Dinglin performed the validation and reliability assessments of the scale among Chinese adolescents (22). The internal consistency of the DERS was considered adequate, supported by a Cronbach's *α* coefficient of 0.96. Furthermore, a Pearson correlation coefficient of 0.76 and a Kaiser-Meyer-Olkin value of 0.948 were reported for the DERS. DERS items are rated on a scale ranging from 1 (almost never) to 5 (almost always). A higher score indicates more severe problems in regulating emotions.

### Statistical analysis

The data were evaluated using the Statistical Package for Social Sciences (SPSS) version 17.0 for Windows. Prior to analysis, missing values, outliers, and normalcy were examined. The data were coded, cleaned, and analyzed using descriptive and inferential statistics. Descriptive statistics, including means, standard deviations, and frequencies, are utilized to describe the sample characteristics and the prevalence of DEBs. The Mann‒Whitney U-test and Kruskal‒Wallis H test were used to evaluate the associations between DEBs in children and adolescents with T1D and sociodemographic variables and the severity of DEBs. A *p* value < 0.05 was considered to indicate statistical significance. Effect sizes were interpreted based on Cohen's guidelines (23), where correlation coefficients between 0.10 and 0.29 indicate a small association, values between 0.30 and 0.49 represent a medium association, and values of 0.50 and above represent a large association.

## Results

### Survey respondent characteristics

Of the 135 individuals who were invited to complete the DEPS-R, 128 agreed to participate, with 7 being eliminated because provided the same answer, ultimately yielding an impressive response rate of 94.81%. The average age of the participants was 11.63 ± 2.27 years. A total of 77.34% of the patients (*n* = 99) were female, and 82.03% (*n* = 105) had been diagnosed with diabetes for less than three years. A total of 20.31% of the patients (*n* = 26) had lipohypertrophy, and 78.91% (*n* = 101) had fingertip blood glucose data. A total of 74.22% (*n* = 95) of the patients were using insulin pens. Table 1 shows the participants' sociodemographic and clinical characteristics.

**Table 1:** Characteristics of the participants (*N* = 128).

Variable	*N* (%)	DESP-R < 20 (*n* = 67)	DESP-R ≧ 20 (*n* = 61)	Value	*P*
Age (years)		11.42 ± 2.38	11.85 ± 2.128	−1.084	0.280
Gender (%)				1.345	0.181
Male	29 (22.66)	14 (20.90)	15 (24.59)		
Female	99 (77.34)	53 (79.10)	46 (75.41)		
BMI (%)				0.698	0.706
Under	17 (13.28)	10 (14.93)	7 (11.48)		
Normal	85 (66.41)	45 (67.16)	40 (65.57)		
Overweight or obese	26 (20.31)	12 (17.91)	14 (22.95)		
Diabetes duration (years)				0.726	0.469
<3	105 (82.03)	57 (85.07)	48 (78.69)		
≥3	23 (17.97)	10 (14.93)	13 (21.31)		
One-child Family (%)				0.502	0.478
Yes	46 (35.94)	26 (38.81)	20 (32.79)		
No	82 (64.06)	41 (61.19)	41 (67.21)		
Medical expenses payment (%)				0.536	0.464
Self-paying	38 (29.69)	18 (26.87)	20 (32.79)		
Medical insurance	90 (70.31)	49 (13.43)	41 (67.21)		
Lipohypertrophy (%)				0.072	0.789
Yes	26 (20.31)	13 (19.40)	13 (21.31)		
No	102 (79.69)	54 (80.60)	48 (78.69)		
Ways of monitor blood glucose (%)				0.656	0.418
Fingertip blood glucose	101 (78.91)	51 (76.12)	50 (81.97)		
Continuous glucose monitoring	27 (21.09)	16 (23.88)	11 (18.03)		
Primary caregiver (%)				1.118	0.290
Parents	115 (89.84)	62 (92.54)	53 (86.89)		
Grandparents	13 (10.16)	5 (7.46)	8 (13.11)		
Education level of primary caregiver (%)				4.708	0.095
Junior high and lower	68 (53.13)	34 (50.75)	34 (55.74)		
Senior high school or above	29 (22.65)	20 (29.85)	9 (14.75)		
University and above	31 (24.22)	13 (19.40)	18 (29.51)		
Total income (%)				1.168	0.558
<4,999	54 (42.19)	27 (40.30)	27 (44.26)		
5,000∼7,999	50 (39.06)	29 (43.28)	21 (34.43)		
>8,000	24 (18.75)	11 (16.42)	13 (21.31)		
Insulin administration mode (%)				0.086	0.769
Insulin pump	33 (25.78)	18 (26.87)	15 (24.59)		
Insulin pen	95 (74.22)	49 (73.13)	46 (75.41)		
Total DEPS-R score, mean (SD)		11.15 ± 5.19	25.07 ± 4.73	−15.87	**<**0.001
Total DERS score, mean (SD)		65.49 ± 16.88	79.77 ± 22.91	−4.038	**<**0.001

### Participants' diabetes eating problem survey-revised scores

The total average DEPS-R score was 17.78 ± 8.56 points, with a theoretical range of 0∼5. A total of 61 participants scored above the DEPS-R threshold of 20 points, with a prevalence rate of 47.66%. The distribution of DEPS-R scores varied based on sex, with a greater proportion of girls (77.34%) experiencing eating problems than boys (22.66%). Among the girls, 73.77% had a DEPS-R score indicating eating problems. In total, 25.78% of the patients exhibited a mild eating problems, 26.56% demonstrated moderate eating problems, and 47.66% displayed severe eating problems. No statistically significant differences were observed in terms of age, sex, parental education level, insulin delivery system, or T1D duration between patients with and without eating problems according to the DEPS-R score.

### Difficulties in emotion regulation among the participants

The total average DERS score was 72.3 ± 21.15 points, with a theoretical range from 1∼5. In the group with a positive DEPS-R screening result, the highest mean scores were reported in the strategies and nonacceptance domains, followed by the awareness, impulses, goals and clarity domains. The total scores on the DEPS-R and its subscales, including the emotional awareness, clarity, acceptance, strategies, impulses, and goals subscales, were found to be significantly greater in individuals who had a DEPS-R score indicating eating problems than in individuals who had a score indicating no eating problems (*P* < 0.05).

### Association between the DEPS-R and DERS scores

**Figure 1:**
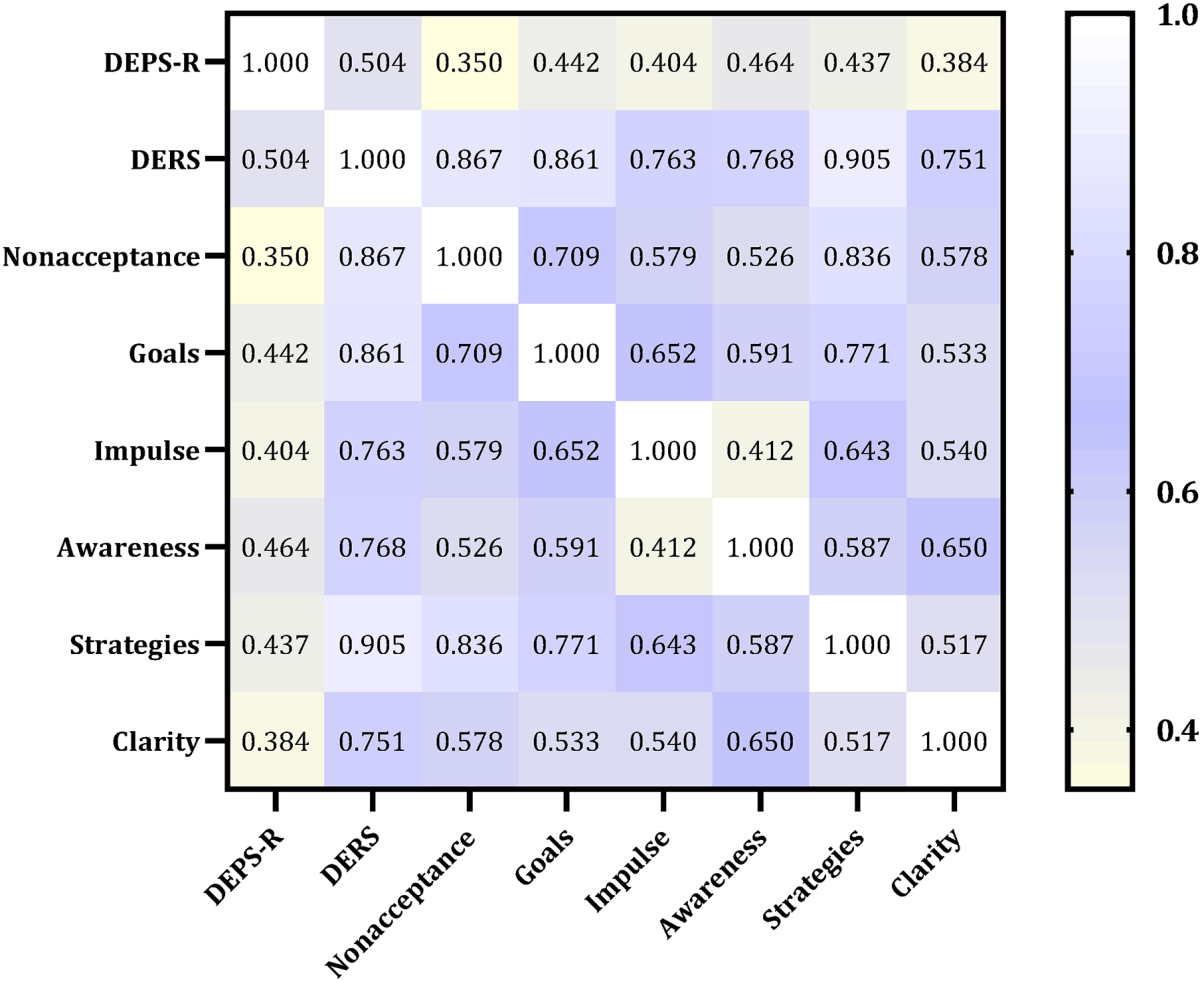
Correlation between the DEPS-R and the DERS scores.

Figure 1 presents the correlation between the DEPS-R and the DERS scores. A significantly positive correlation was observed between the DERS dimension scores and the DEPS-R scores, which indicated that individuals with higher scores in emotional regulation are more likely to exhibit eating behavior problems.

### Factors associated with diabetes eating problems

According to Pearson correlation analysis, the total score of DEPS-R was significantly correlated with DERS, nonacceptannce, goals, impulse, awareness, strategies, and clarity. A stepwise linear regression analysis was performed to measure which variables affect a DEB-risk in children and adolescents with T1D (Table 2). The strongest combined determinants of the DEB risk were the DERS scores (*β* = 0.327, SE = 0.062, *t* = 5.306, *p* < 0.001) and the Nonacceptance score (*β* = −0.701, SE = 0.305, *t* = −2.300, *p* = 0.023), yielding an R of 0.533 (explained variance 27.2%) (Table 2).

**Table 2:** Stepwise linear regression analysis.

Variables	Unstandardized Coefficients		*t*	*P* value	95% CI	*R*	Adj *R*^2^
	*β*	SE					
1. (Consent)	3.035	2.347	1.293	0.198	−1.611∼7.680	0.504	0.248
DERS	0.204	0.031	6.543	0.000	0.142∼0.266		
2. (Consent)	1.496	2.403	0.622	0.535	−3.261∼6.253	0.533	0.272
DERS	0.327	0.062	5.306	0.000	0.205∼0.449		
Nonacceptance	−0.701	0.305	−2.300	0.023	−1.303 to −0.098		

DEPS-R, the diabetes eating problem survey-revised scores; DERS, the difficulties in emotion regulation scale.

## Discussion

Emotional regulation challenges and the occurrence of DEBs are two significant obstacles that can emerge for children and adolescents, potentially impacting their self-care practices and glycemic control in a detrimental manner. Consequently, this study aims to evaluate the frequency of DEBs among children and adolescents diagnosed with T1D while also exploring the correlation between these actions and DERS scores. Consistent with our hypothesis, within our sample of children and adolescents with T1D, issues concerning emotion regulation were more prevalent among individuals exhibiting DEBs than among those with minimal to moderate levels of such behaviors. Thus, there is a significant relationship between emotion regulation problems related to and DEBs in adolescents with T1D.

No significant differences were detected in the chi-square analyses performed to investigate differences between the two groups in terms of sex, age, BMI, insulin administration mode, or T1D duration, which is similar to the findings of Yilmaz KH et al. (24). However, further large studies are needed to confirm whether DEBs are more common in girls than boys, as suggested by other research (25). This difference could be attributed to cultural variances or limitations in sample size.

In the present investigation, 47.66% of the included participants had a positive screening result for DEBs. These findings align with data obtained from cross-sectional studies conducted in children and adolescents with T1D across various global populations, with a rate of 54.6% reported by Zhou Meijing (26), 30.1% reported for Greek populations (27), and 37.7% reported for Italian populations (28). However, these finding were slightly greater than those of a systematic review of eDEBs in adolescents with T1D conducted by Zhang Yu et al. (34%) (29). The reasons for these differences may be differences in demographic data, study locations, and survey methods. However, the overall data results remain high, indicating a high incidence of DEBs in children and adolescents with T1D.

Several potential diabetes-related factors may clarify the susceptibility of individuals with T1D to DEBs, especially children and adolescents. Initially, the event of hypoglycaemia, wherein the body instinctively requires nourishment to elevate blood sugar levels, could lead to a bout of excessive food consumption (30). In comparison to adults with T1D children and adolescents with T1D demonstrate a relatively diminished capacity to regulate their food intake, consequently increasing their susceptibility to developing DEB (31). Moreover, after diagnosis, the absence of insulin typically results in weight loss caused by glucosuria and catabolism, which is subsequently succeeded by a swift weight increase after commencing insulin therapy (25). This unfavourable weight gain has the potential to trigger the emergence of unhealthy eating patterns and decrease compliance with insulin treatment (32).

Currently, the primary focus in research concerning DEBs in adolescents diagnosed with T1D is identifying the risk factors associated with their clinical characteristics. Nonetheless, importantly other factors linked to the onset of T1D could enhance the likelihood of developing DEBs, such emotions regulation. Children and adolescents with uncontrolled emotion regulation are more likely to overeat or steal additional food and subsequently develop DEBs, which can lead to serious complications, such as diabetic ketoacidosis (33, 34). The occurrence of emotion dysregulation is reported across individuals with various diagnoses. This serves as the crucial focus of interventions aiming to address emotion dysregulation. However, studies on the topic of emotion regulation in Chinese children and adolescents diagnosed with T1D, as well as investigations examining the use of the DERS, are lacking.

The results of this study showed that the DEPS-R scores of children and adolescents with T1D were significantly different in terms of emotional regulation, and DEBs were correlated with emotional regulation and scores in all dimensions. This means that the higher the score for DEBs in children and adolescents is, the lower their level of emotional regulation ability is; that is, the greater the score the emotional regulation is (35). Yilmaz KH et al. discovered a significant link between DEBs and challenges accessing strategies for regulating emotions, as well as difficulties in effectively regulating one's own emotional responses (24). In addition, Walenda A et al. reported that individuals who had negative emotions before eating were at greater risk of overeating during their next meal (36).

For children and adolescents with T1D, self-management may be challenging, and the self-acquiring emotional regulation strategies is difficult for these individuals; additionally, their self-awareness and self-control are low, and they are prone to DEBs (37). Moreover, these findings imply that individuals with diabetes can effectively manage their emotions by enhancing their emotional regulation ability. Consequently, they can successfully address eating behavior issues (38). However, children and adolescents are in a period of rapid physical and psychological development, and psychological aspects are becoming more sensitive (39). In the face of negative emotions, because their emotion regulation methods are immature, children and adolescents have no systematic concept of how to correctly address negative emotions (40). Emotional fluctuations interfere with individual cognitive control, thus promoting the development of DEBs in children (36).

This study has strengths. A notable advantage of this study is that it specifically focused on a sample comprising individuals aged 8–16 years. This sample selection approach enhanced the ability to comprehensively understand the connection between issues related to emotion regulation and the development of disordered eating patterns in children and adolescents diagnosed with T1D. Second, difficulties in emotion regulation may be an indicator ofDEBs in patients (8∼16 years old) with T1D.

Our study has several limitations. First, it should be noted that the cross-sectional design employed in this research does not allow us to make inferences about the directionality of the associations between the variables under investigation. Therefore, it is imperative to conduct prospective studies to address this issue. Second, it is essential to acknowledge that the DEPS-R is a self-report tool, which may introduce biases such as self-report bias and recall bias. Furthermore, the questionnaires were completed by the children and adolescents with diabetes in this study, which may be slightly prejudiced compared to what their parents would have noticed. Consequently, caution must be exercised when interpreting the results obtained using this instrument. Additionally, the generalizability of our findings may be influenced by selection bias, given that our sample consisted solely of adolescents with T1D from China. It is unclear whether these results would hold true among adolescents with T1D from other countries. Consequently, future studies, including longitudinal studies, are needed to gain a better understanding of the associations between emotion regulation problems and DEBs. It is also crucial to conduct long-term follow-ups to elucidate the determinants of eating disorders in this population. Moreover, such follow-ups will provide valuable insights for informing the development of effective interventions aimed at prevention.

## Conclusions

In brief, our study revealed that there is a link between challenges in managing emotions and unhealthy eating habits in children and adolescents (aged 8–16years) with T1D. By gaining a deeper understanding of the connection between emotion regulation difficulties and DEBs in individuals with T1D, we can pave the way for the development of more efficient treatments for this specific population. Moreover, if healthcare professionals are well informed about potential complications related to emotion regulation, they can proactively identify and address any early signs of disordered eating behaviors, ensuring that they are addressed promptly and before they become entrenched and harder to manage. Hence, it is crucial for healthcare providers in clinical settings to be attentive to the adverse consequences associated with DEBs and emotional regulation. The regular assessment of adolescents diagnosed with T1D is pivotal, as is timely intervention is needed to avoid progression toward clinically relevant psychiatric disorders or clinical complications in late adolescence and adulthood.

## Data Availability

The original contributions presented in the study are included in the article/Supplementary Material, further inquiries can be directed to the corresponding author.
